# Outdoor Physical Activity and Youth Mental Well-Being: A Narrative Review with Mountain Biking as an Illustrative Case

**DOI:** 10.3390/sports14050166

**Published:** 2026-04-22

**Authors:** Katherine Mommaerts, Ruby Johnson, Sydney Joy Varner, Nathalia Marchese

**Affiliations:** 1 Department of Social Work, Northern Arizona University, Flagstaff, AZ 86011, USA; rjj85@nau.edu (R.J.); sjv85@nau.edu (S.J.V.); 2 Department of Biological Sciences, Northern Arizona University, Flagstaff, AZ 86011, USA; nm2567@nau.edu

**Keywords:** youth mental health, outdoor physical activity, mountain biking, resilience

## Abstract

This narrative review synthesizes existing literature on outdoor physical activity and youth well-being, with mountain biking considered as an illustrative example of a high-engagement, nature-based activity. A comprehensive literature search was conducted across multiple academic databases, including Academic Search Complete, MEDLINE, APA PsycINFO, CINAHL Plus, SocINDEX, ERIC, and additional hand searches in Google Scholar and Web of Science. Seventeen studies met the inclusion criteria and were analyzed using an iterative thematic approach. Three primary themes emerged: resilience, mood and emotional well-being, and social connectedness. Across studies, outdoor physical activity was associated with improvements in self-efficacy, stress reduction, and peer relationships. However, most studies examined outdoor activity broadly, with limited evidence specific to mountain biking. While prior literature suggests that biological and psychosocial processes (e.g., engagement with nature, social interaction, and perceived competence) may underlie these associations, these mechanisms were not directly tested in most included studies. Findings should therefore be interpreted as indicative of associations rather than causal effects. Overall, outdoor physical activity represents a promising, accessible approach for supporting youth well-being. Future research should further examine activity-specific impacts, including mountain biking, and prioritize longitudinal and experimental designs to better understand mechanisms and long-term outcomes.

## 1. Introduction

The emotional and psychological well-being of youth is a pressing public health priority. Mental health disorders such as anxiety, depression, and stress-related conditions are now recognized as significant public health challenges, disproportionately affecting marginalized youth who face compounded mental health risks due to socioeconomic, racial, and environmental disparities [1]. Currently, an estimated 166 million youth worldwide experience a mental health condition, with anxiety and depressive disorders among the most prevalent [1]. In the United States, rates of youth anxiety, depression, and emotional distress have also increased in recent years [2]. These trends are particularly concerning because adolescence represents a critical period of psychological and emotional development, during which unresolved mental health challenges may have long-term consequences extending into adulthood [3]. Physical activity has been increasingly recognized as an important protective factor for youth mental health, offering both physiological and psychosocial benefits that may support emotional well-being during adolescence [4].

The transition from youth to adulthood is marked by profound biological, psychological, and social changes, which are further shaped by structural inequities. Marginalized youth—including those from under-resourced and low-income backgrounds, racialized communities, and individuals with disabilities—often experience limited access to mental health resources, chronic stressors, and systemic barriers that exacerbate emotional distress [5]. During puberty, individuals navigate shifts in physical health, identity formation, and cognitive maturation while also contending with economic instability, discrimination, and adverse social conditions that elevate mental health risks [6]. These challenges underscore the importance of coping strategies that support overall well-being and resilience during this critical developmental stage. As biological and environmental factors shape youth development, identifying factors that support well-being is essential.

### 1.1. Physical Activity and Mental Health

While physical activity is widely recognized for its physiological benefits, emerging research highlights its significant impact on youth well-being. Outdoor physical activity has been linked to improved psychological health, including reductions in anxiety, stress, and social isolation [7]. These benefits have been identified to operate through neurobiological and psychosocial pathways, including potential neurobiological response (e.g., neuroplasticity and neurotransmitter activity), and psychosocial processes such as mastery, autonomy, and social interaction, although these mechanisms are not directly tested in most studies [8].

Outdoor recreation activities, including hiking, mountain biking, skiing, and other recreational sports, may offer distinct psychological benefits, particularly for marginalized youth who often face barriers to traditional mental health services [9]. Participation in nature-based activities has been associated with lower stress levels, increased self-efficacy, and stronger social connectedness, making these activities a promising approach for enhancing youth well-being and resilience [10]. While several reviews exist on ‘green exercise’ (i.e., physical activity performed in natural environments), there is a lack of synthesis specifically linking high-engagement outdoor sports like mountain biking to resilience frameworks in marginalized youth. Recognizing the multifaceted nature of mental health, nature-based physical activity represents a potentially accessible and holistic strategy for fostering resilience among youth. Previous reviews have examined the relationship between physical activity and youth mental health; however, fewer studies have focused specifically on outdoor recreation or high-engagement activities such as mountain biking.

### 1.2. Theoretical Frameworks

To understand the complex relationship between physical activity, well-being, and youth development, this study draws upon two key theoretical frameworks: The biopsychosocial model and the ecological systems theory. The biopsychosocial model is uniquely suited for this review as it accounts for the multifaceted nature of youth development [11]. In the context of outdoor recreation, the biological component involves the physiological response to exercise and nature immersion (e.g., cortisol reduction). The psychological component addresses the individual’s internal experience of ‘flow’ and self-efficacy gained through mastering technical biking skills. Finally, the social component recognizes the peer and community networks formed through group-based sport participation. Together, these factors interact to foster a more resilient psychological profile than sedentary or solo activities might provide [11]. Furthermore, the ecological systems theory provides the structure for understanding how environmental contexts influence resilience [12]. For example, mountain biking programs serve as a vital microsystem, a setting where youth experience direct social interactions and skill development. This review also considers the macrosystem, exploring how broader cultural shifts and initiatives influence the availability and social acceptance of outdoor interventions. By examining these overlapping systems, the review identifies how outdoor activity acts as a protective buffer across multiple levels of a young person’s environment.

### 1.3. Purpose of Study

While the broad benefits of outdoor physical activity are well-documented, there is an increasing need to align research with the practical expansion of community-based sports programs. This review was informed by a community-based initiative that prioritizes outdoor recreation, specifically mountain biking, as a strategy for supporting youth development. In the literature on youth outdoor recreation, many studies do not measure clinical mental health disorders directly but instead examine related psychosocial outcomes. Using the aforementioned frameworks, they provide the lens through which the following literature is synthesized: the biopsychosocial model informs our analysis of individual mood and resilience, whereas the ecological systems theory guides our understanding of social well-being and community-based implications. This study examines how outdoor physical activity is associated with youth resilience, mood and well-being, and social connectedness, with mountain biking considered as an illustrative example within the broader context of nature-based activity. This analysis contributes to the growing body of research examining nature-based interventions as accessible strategies for supporting youth well-being.

## 2. Materials and Methods

A narrative review was conducted to examine the relationship between outdoor physical activity and youth well-being. This review was designed to synthesize existing research on outdoor recreation and youth mental health, with a particular focus on nature-based activities such as mountain biking. This study is a narrative literature review. While systematic search strategies were used to ensure a comprehensive scope, it does not function as a formal systematic review or meta-analysis and therefore does not include a formal risk-of-bias assessment.

### 2.1. Community Advisory Board

A community advisory board (CAB) was established through a partnership between Northern Arizona University (NAU) and Flagstaff Youth Riders (FLYRS). FLYRS is a Flagstaff, Arizona-based nonprofit organization that provides youth with mountain biking opportunities regardless of background or economic barriers. The CAB included representatives from seven youth mountain biking programs across Arizona, Colorado, and New Mexico, as well as a small research team from NAU.

The CAB met three times over a four-month period. The CAB provided context on mountain biking programs in the Southwest and assisted with aligning the research agenda with community-identified priorities. The goal of the CAB was to identify community and environmental factors that influence youth development, with a particular focus on promoting positive outcomes through outdoor physical activity, specifically mountain biking. Additionally, the CAB helped identify key outcomes to include in the FLYRS research agenda related to youth mental health and physical activity. One outcome of the CAB process was the development of this literature review. CAB members provided feedback and recommendations on the scope and relevance of the review to ensure alignment with community priorities and lived experiences.

### 2.2. Data Collection

A literature search was conducted to identify studies examining how outdoor physical activity influences youth mental well-being. Electronic databases, including Academic Search Complete, MEDLINE, APA PsycINFO, APA PsycARTICLES, CINAHL Plus with Full Text, SocINDEX with Full Text, Education Full Text (H.W. Wilson), and ERIC, were searched. Additional hand searches were conducted using Google Scholar and Web of Science to identify relevant studies that may not have been captured through database searches. In consultation with a research librarian, the following keywords were developed and used in various combinations: youth, youth mental health, outdoors, time outdoors, physical activity, mountain biking, green exercise, green environment, and positive youth development. The search strategy employed Boolean operators (AND, OR) to ensure both breadth and specificity in the results. Specifically, terms for the target population (“youth” OR “adolescent”) were combined with the primary intervention (“mountain biking” OR “outdoor physical activity” OR “green exercise”) and the identified psychological outcomes (“positive youth development”). This specific combination of keywords was chosen to isolate studies that move beyond general ‘green exercise’ to specifically intersect high-adventure, high-engagement sports with psychological coping mechanisms and resilience frameworks. The search included peer-reviewed articles (2014–2024) published in English focusing on youth aged 10–18. Studies were considered relevant for inclusion if they (1) focused on youth aged 10–18, (2) examined outdoor or nature-based physical activity, and (3) reported psychosocial or mental health-related outcomes (e.g., resilience, mood, stress, or social well-being). A total of 34 records were initially identified. Following a title and abstract screening for relevance to youth-specific outcomes and nature-based activity, 17 studies were selected for full-text review and inclusion. Studies were further categorized based on activity type. Studies were classified as mountain biking-specific when mountain biking was the primary activity examined, while all other studies were categorized as general outdoor physical activity. This distinction was used to support interpretation of findings and to avoid overstating activity-specific conclusions. The selection focused on studies that provided empirical data, whether qualitative, quantitative, or mixed-methods, on the psychosocial impacts of outdoor engagement. Themes were developed through an inductive, iterative analysis of the included studies. Initial thematic groupings were identified based on patterns observed across study findings and were subsequently refined in consultation with the CAB to ensure contextual relevance and practical applicability. Articles examining multiple racial and ethnic populations were incorporated, and studies conducted outside of the United States were included to provide a broader understanding of outdoor activity and youth mental health outcomes. Articles were excluded if they focused primarily on adults or fell outside the defined publication date range. Three researchers conducted the search and review process in consultation with a research librarian between Fall 2024 and Spring 2025. The CAB contributed to the interpretation and contextualization of findings but did not influence study inclusion decisions or the primary analytic process.

## 3. Results

This review identified 17 studies supporting the benefits of outdoor physical activity, with mountain biking programs representing a limited but illustrative subset of the literature (see Table 1). 

Guided by the priorities of the CAB and framed through the biopsychosocial model and ecological systems theory, three primary themes emerged regarding youth well-being: resilience (psychological coping and self-efficacy; 6 studies), mood and emotional well-being (affective states and stress reduction; 9 studies), and social well-being (peer relationships and social connectedness; 6 studies). Some studies addressed outcomes across multiple themes; therefore, the sum of studies per theme exceeds the total of 17 reviewed. Youth who participate in physical activity may experience improvements in factors associated with mental health, such as reduced psychological distress and improved mood; however, youth engaging in organized sports have an increased risk for developing anxiety, alcohol use, and bulimia [25]. Although physical activity may not directly reduce the likelihood of clinical mental health diagnoses, many studies suggest that it influences related factors such as self-esteem, sleep quality, stress levels, and social connectedness [13,21]. Physical activity can be beneficial to factors that influence mental health and has shown increased positive outcomes when paired with the outdoors.

Youth who participate in outdoor activities demonstrated greater improvements in psychological well-being compared with those participating in indoor activities [24]. One study found that 22.1 percent of youth participants said the outdoors relieves stress or anxiety, and 51.6 percent reported that being in nature makes them feel calm [23]. Engaging in outdoor physical activity in youth may continue into adulthood. Adults also report that time spent in nature compared to inside or in an urban setting results in decreased anxiety, rumination, and increased sense of self and connection and care with the human and non-human world [14]. These findings suggest that when pairing physical activity with being outdoors, there is an increase in positive benefits in youth mental health that could impact later life.

### 3.1. Resilience

Outdoor physical activity has been shown to promote the development of life skills and characteristics in youth; growth in resilience was a common theme found throughout various articles. For example, during the COVID-19 pandemic, youth who frequently engage in outdoor activity were shown to maintain positive well-being through resilience [17]. Resilience encompasses several characteristics associated with positive youth development, including strength, confidence, capability, and motivation. Participation in outdoor physical activity has been associated with increased independence, self-assurance, and autonomy [13,15]. One study focused on youth outdoor bicycling stated that youth had increased levels of responsibility for gear and safety, self-confidence, independence, and feelings of freedom after participating in the program [26].

Evidence suggests an association between outdoor bicycling and increased independence. For instance, participants in the Momentum Bicycle Clubs (MBC) demonstrated growth in responsibility, though results rely heavily on self-reported improvements [26]. Though mentorship was a part of MBC’s program, researchers claimed the group would not have reached the same level of success without the aspect of bike riding [26]. Participants also experienced an increase in confidence as they were able to strengthen stamina, explore new areas, and experience freedom [26]. Researchers found that the bike itself offered growth in responsibility, confidence, and health [26]. Taking care of equipment contributed to increased responsibility, and the improvement of bicycle riding and navigational skills contributed to self-confidence and physical health [26]. Overall, outdoor physical activity has been shown to be a beneficial way to foster resilience in youth. In addition to resiliency, outdoor physical activity impacts youths’ mood [27]. Longitudinal evidence further suggests that regular physical activity during adolescence supports well-being through mechanisms such as improved self-esteem, perceived competence, and social connectedness, which are key components of resilience development [16].

### 3.2. Mood and Emotional Well-Being

In this review, mood and emotional well-being refer to short-term emotional states, such as stress reduction, feelings of calm, or enjoyment associated with participation in an outdoor activity. These outcomes differ from clinical mental health diagnoses or long-term psychiatric conditions. Outdoor activity is associated with lower reported stress levels [14]. One youth noted, “outside there is no stress [13],” though this reflects an immediate emotional state rather than a clinical reduction in mental health symptoms. Similarly, afterschool programs that incorporate outdoor physical activity provide students with opportunities to disengage from academic stress [13].

Stress is a common factor influencing youth emotional well-being; engagement in enjoyable, social activities primarily impacts one’s immediate mood and stress levels. Participants from a bicycling study reported that bicycling reduces stress, promotes relaxation, increases social interactions, and is a fun outdoor activity [26]. Biking also increases feelings of freedom and autonomy, which positively shift short-term mood and contribute to overall subjective well-being [26].

### 3.3. Social Well-Being

Social well-being, social skills, relationships, and peer connections are vital in youth mental health. Being outdoors with a friend results in higher levels of mood restoration compared to being alone, and an increase in social interactions; participants reported that outdoor physical activities provide valuable opportunities for peer interaction [24]. Participants from a study where youths were part of a group in an outdoor physical activity-focused afterschool program reported forming new friendships, and 42% of the participants stated that before the program, they would spend time alone in their rooms after school and now spend time with new friends [13]. The youth who formed new friendships also discussed that they have more people they trust, have more fun, and are not as lonely [13]. In a study with 28 youth participants who engaged in a mountain biking program, all 28 members stated that one of the main things they enjoy about mountain biking is being a part of a team and feeling supported by their peers [15]. Some outdoor adventure programs aim to use the combination of nature and physical activity as an intervention for youth development. Participants reported living in nature to be a “disorienting dilemma” which facilitated self-reflection and the opportunity to grow in confidence by working as a community [28]. Outdoor physical activity impacts youths’ mental health with main results in character building, including resilience, mood and social well-being.

## 4. Discussion

The three themes identified in this review, resilience, mood and emotional well-being, and social well-being, suggest the multifaceted ways outdoor physical activity may support youth mental health. While the evidence presented strongly supports outdoor activity broadly, the specific evidence for mountain biking remains an illustrative subset of the nature-based literature. Across studies, engagement in nature-based recreation was consistently associated with improvements in resilience, mood, and social connectedness. The benefits are particularly relevant for youth, who often face heightened socioeconomic stressors, limited access to traditional mental health services, and fewer opportunities for recreational activities. These findings suggest that outdoor recreation may support youth development through multiple mechanisms, including physical activity, social interaction, and engagement with the outdoor environment. Importantly, the evidence synthesized in this review primarily reflects outdoor physical activity broadly, with mountain biking representing a smaller subset of the available literature.

The findings from this literature review suggest outdoor physical activity may support several dimensions of youth well-being, particularly resilience, mood regulation, and social connectedness. While there is substantial evidence supporting the link between physical activity and mental well-being, research findings are not uniform. For example, some studies indicate that regular engagement in physical activity reduces social anxiety and strengthens self-esteem [21], whereas other studies found no significant relationship between physical activity and the reduction of clinical mental health disorder symptoms [29]. Despite these discrepancies, longitudinal data suggest that high levels of physical activity in early adolescence are associated with lower emotional distress in later years [16,21]. This supports the hypothesis that physical activity may function as a protective factor against long-term mental health challenges. Proposed explanations in prior literature include neurobiological and psychosocial processes (e.g., neuroplasticity, neurotransmitter activity, and enhanced self-efficacy); however, these mechanisms were not directly tested in most of the studies included in this review and should therefore be interpreted as theoretical rather than empirically established within this evidence base [30,31,32]. Additionally, current data does not support a causal claim that mountain biking “improves” clinical outcomes.

The unique benefits of outdoor activity, particularly mountain biking, further highlight the role of nature-based recreation in mental health interventions. Mountain biking may represent a unique form of outdoor recreation that integrates physical exertion, skill development, risk management, and environmental engagement; however, the evidence specific to mountain biking remained limited within the current literature and is best interpreted as illustrative of broader patterns observed in outdoor physical activity research. Research on nature exposure and mental well-being consistently demonstrates that time spent in green spaces enhances self-perceived competence, emotional stability, and overall psychological development [20]. Participants in outdoor adventure programs report reduced stress, increased emotional resilience, and greater feelings of connection to their environment [28]. However, an important moderating factor is the presence of peers or social support systems; youth who engage in outdoor physical activities with others experience greater psychological benefits than those who do so alone [24]. This reinforces the importance of structured, community-based programs that integrate peer support and mentorship, particularly for youth from underserved communities who may lack access to independent outdoor recreation.

A growing body of research has introduced the concept of “green exercise,” referring to physical activity conducted in natural environments [22]. Evidence suggests that outdoor physical activity may be more effective at reducing stress, anxiety, and depressive symptoms than exercise performed in urban or artificial settings [18,33]. Among youth, engagement with green spaces has also been associated with lower emotional distress, improved peer relationships, and fewer behavioral difficulties [34]. These findings are particularly relevant to mountain biking, which combines physical exertion, environmental engagement, and skill development, all of which may contribute to resilience-building among youth.

Given these findings, mountain biking programs tailored for marginalized youth may represent an accessible strategy for supporting youth well-being, particularly through improvements in resilience, mood regulation, and peer connectedness. Community-based initiatives that provide affordable equipment, safe spaces, and mentorship opportunities can help overcome barriers to participation, such as cost, transportation, and access to green spaces. Additionally, integrating mountain biking into youth development and mental health programs could provide a sustainable, non-stigmatizing approach to mental health support, particularly for those who may not seek traditional therapy.

The findings of this review align closely with the biopsychosocial model and ecological systems theory, providing a theoretical basis for how outdoor physical activity influences youth development. From a biopsychosocial perspective, outdoor physical activity may engage biological, psychological, and social processes. For example, physical exertion has been associated with neurotransmitter activity, while participation in structured activities may support self-efficacy and social connection. Within this framework, mountain biking can be understood as one example of an activity that may engage these processes, although these mechanisms were not directly evaluated in the included studies. Simultaneously, the psychological concept of ‘flow’ experienced during skill-based activities such as technical riding may contribute to perceived self-efficacy, while the social nature of group-based participation may support peer connection and emotional well-being. Furthermore, applying ecological systems theory, mountain biking programs can be conceptualized as operating within the microsystem by providing structured opportunities for peer interaction and adult mentorship outside of school or home settings. When supported by broader policy and community investment, these programs may also reflect influences at the macrosystem level by shaping cultural institutional support for nature-based interventions. Taken together, these frameworks offer a way to interpret how outdoor recreation may contribute to youth well-being across multiple ecological levels, though these pathways should be understood as theoretical rather than empirically established within the reviewed studies.

### 4.1. Limitations

Several limitations should be considered when interpreting the findings of this literature review. Many of the included studies relied on self-reported data from youth participants, teachers, mentors, or program facilitators, which may introduce response or social desirability bias. Additionally, much of the existing literature utilizes cross-sectional designs, limiting the ability to assess long-term mental health outcomes associated with outdoor physical activity. The limited availability of longitudinal studies further restricts conclusions about whether the observed benefits of outdoor physical activity persist over time.

There are also limitations related to the design of this review. This study was conducted as a narrative review rather than a systematic or scoping review and therefore did not follow formal review protocols such as structured study screening procedures or standardized quality assessment tools. As a result, it is possible that some relevant studies were not captured despite the use of multiple databases and keyword search strategies. Additionally, while this narrative review synthesizes a broad range of evidence, the general quality of the literature in this field presents certain methodological considerations. Many of the included studies relied on relatively small, localized sample sizes, which may limit the generalizability of the findings to broader youth populations. Additionally, while the review incorporated diverse geographic contexts, the majority of research remains centered in high-income countries, suggesting a need for more diverse representation to understand how different socioeconomic and cultural factors influence the efficacy of outdoor interventions.

Additionally, many studies measured outcomes related to mood, stress, or perceived well-being rather than clinical mental health diagnoses, which limits conclusions about the direct impact of outdoor physical activity on psychiatric conditions. The limited number of mountain biking-specific studies further restricts the ability to draw activity-specific conclusions, reinforcing the need to interpret findings within the broader context of outdoor physical activity. While these findings are encouraging, the reliance on cross-sectional and self-reported measures also suggests that these findings should be interpreted cautiously when considering clinical or intervention-based applications. Addressing this gap may inform the development of inclusive, evidence-based interventions that incorporate outdoor recreation into youth mental health promotion.

Despite these limitations, the review provides a synthesis of existing research on outdoor physical activity and youth well-being and highlights important areas for future research, particularly related to nature-based recreation and activities such as mountain biking.

### 4.2. Implications for Practice and Future Research

These findings suggest that structured outdoor recreation programs may represent a promising strategy for promoting both physical activity participation and well-being among youth. While existing literature supports the mental health benefits of outdoor physical activity, more research is needed to explore how outdoor physical activity, specifically mountain biking, could contribute to psychological resilience in marginalized youth. Mountain biking represents a unique form of outdoor recreation that integrates physical exertion, skill development, risk management, and environmental engagement. Future programs might consider utilizing outdoor activity in nature as a tool to support positive emotional states, such as improved mood and self-esteem.

Cultural and economic factors may produce conflicting results in the relationship between physical activity and mental health [35]. The association between physical activity and well-being seems to be consistent across Western and high-income Eastern countries, but there is little research on middle-income countries and their youth [35]. Low levels of activity may be due to cultural or economic factors. For example, a deficit of playgrounds in Pakistan may be a contributor to low levels of physical activity [35,36]. The study of green play time amongst Spanish youth found that generally, children of households with parents of lower education, facing unemployment or single parenting, or of non-Spanish origin had a lower amount of green space playing time, higher SDQ scores, and a larger amount of ADHD symptoms [34]. Future research may benefit from a wider variety of economic statuses by considering the financial, psychological, and sociocultural factors that may impact the frequency of youth engagement in physical activity [37]. When studying the mental health of youth, variables such as socioeconomic status, cultural norms, or familial structures need to be considered.

These findings suggest that schools, community organizations, and youth development programs may consider integrating structured outdoor recreation opportunities as complementary strategies to support youth well-being. High-engagement activities such as mountain biking programs may provide accessible opportunities for physical activity, peer connection, and skill-building that contribute to resilience and emotional well-being, particularly for youth who may face barriers to traditional mental health services.

Though studies have found positive benefits of outdoor physical activity, negative outcomes are still present. Research consistently shows an association between participation in organized sports and alcohol use [25]; there is a direct association between youth engaging in physical activity and the development of alcohol use disorder and bulimia, specifically among youths who engaged in organized sports [25]. Findings have shown that involvement in sports increases alcohol usage but is countered with greater self-esteem [25]. This suggests that future research needs to consider the impacts of organized sports and the negative outcomes of outdoor physical activity.

## 5. Conclusions

The findings of this literature review highlight three primary themes related to the impact of outdoor physical activity on youth well-being: resilience, mood and emotional well-being, and social well-being. Studies examining outdoor physical activity, including a limited number focused on mountain biking programs, suggest that participation in outdoor physical activity can foster these outcomes by promoting responsibility, confidence, and a sense of autonomy among youth participants. Engaging in physical activity within natural environments, such as mountain biking, has been associated with reduced stress and improved psychological well-being.

Outdoor recreation provides a unique opportunity to support youth development while simultaneously strengthening peer relationships and social connectedness. Many studies suggest that it is the combination of physical activity and exposure to natural environments that contributes most strongly to positive youth development. Expanding research in this area, particularly studies that isolate specific activities such as mountain biking, may help clarify activity-specific contributions to youth well-being.

## Figures and Tables

**Table 1:** Characteristics and Thematic Outcomes of Included Studies.

Theme	Study Reference	Key Findings
Resilience	Barfield et al. (2021) [13]	Increased independence, self-assurance, and autonomy through outdoor activity.
	Brent et al. (2021) [14]	Maintained positive well-being through resilience during the COVID-19 pandemic.
	Clanton et al. (2021) [15]	Increased responsibility, self-confidence, and independence in youth bicycling.
	Doré et al. (2020) [16]	Physical activity contributes to mental health via increased self-esteem and social support, reinforcing the biological and social layers of resilience.
	Griffin et al. (2020) [17]	Development of life skills and characteristics needed for success.
	Shanahan et al. (2019) [10]	Outdoor activity as a mechanism for fostering resilience.
Mood and Emotional Well-Being	Amoly et al. (2014) [18]	Association between green space and fewer behavioral difficulties or emotional distress.
	Barfield et al. (2021) [13]	50% of participants reported feeling less stressed while outside.
	Bratman et al. (2015) [19]	Decreased anxiety and rumination in nature compared to urban settings.
	Das & Gailey (2022) [20]	Link between “green exercise” and reduced stress and anxiety symptoms.
	Feng et al. (2024) [21]	Physical activity reduces social anxiety and strengthens self-esteem.
	Han (2017) [22]	Nature exposure enhances emotional stability and psychological development.
	Hossain et al. (2024) [8]	Exercise stimulates neurotransmitters to reduce anxiety and depression.
	Maund et al. (2019) [7]	Nature-based interventions for managing anxiety and depression.
	Zamora et al. (2021) [23]	51.6% of youth reported that nature makes them feel calm.
Social Well-Being	Barfield et al. (2021) [13]	42% of youth moved from isolation to spending time with new friends.
	Clanton et al. (2021) [15]	Bicycling promotes relaxation and increases social interactions.
	Greenwood & Gatersleben (2016) [24]	Higher levels of mood restoration when outdoors with friends vs. alone.
	Griffin et al. (2020) [17]	Youth enjoy being part of a team and feeling supported by peers.
	Maund et al. (2019) [7]	Outdoor activity linked to reductions in social isolation.
	Shanahan et al. (2019) [10]	Nature-based interventions for improving social well-being.

*Note*. *N* = 17. Some studies (i.e., Barfield et al., Griffin et al., and Clanton et al.) contributed evidence to multiple themes within the Section 3.

## Data Availability

Data sharing is not applicable to this article as no new data were created or analyzed during this study.

## Abbreviations

The following abbreviations are used in this manuscript:

CAB	Community Advisory Board
NAU	Northern Arizona University
FLYRS	Flagstaff Youth Riders
MBC	Momentum Bicycle Clubs
